# Resident Perspectives on the Coaching Approach: A Qualitative Analysis of a Longitudinal Coaching Program in an Emergency Medicine Residency

**DOI:** 10.1002/aet2.70111

**Published:** 2025-12-02

**Authors:** Simanjit K. Mand, Corlin M. Jewell, Dana E. Loke, Meinkeng S. Acha‐Morfaw, Collin T. Michels, Benjamin H. Schnapp

**Affiliations:** ^1^ BerbeeWalsh Department of Emergency Medicine University of Wisconsin School of Medicine and Public Health Madison Wisconsin USA

## Abstract

**Background:**

Coaching holds great potential for learners in graduate medical education (GME) as the specialty transitions towards competency‐based medical education (CBME). Coaching has already demonstrated holding great benefits in a variety of learner performance and well‐being metrics. However, there is limited data on learner perceptions of longitudinal GME coaching initiatives.

**Objective:**

To provide insight into learner perceptions of a longitudinal coaching program by conducting focus groups consisting of residents from a single emergency medicine residency.

**Methods:**

Four semi‐structured focus groups were conducted with residents from each post‐graduate year. Audio recordings of focus group discussions were transcribed and a thematic analysis utilizing a constructivist approach was conducted.

**Results:**

We identified five overarching themes: (1) resident understanding of the coach's role, (2) logistical elements that can affect the effectiveness of the coaching relationship, (3) relational factors that contribute to a successful coaching relationship, (4) resident perceptions of discussion topics and goal creation, and (5) future group opportunities for fostering more personal relationships and professional development.

**Conclusion:**

Overall, residents noted several strengths in the longitudinal coaching approach, particularly around feedback reflection and utility, accountability, and goal‐setting. There remain areas for improvement when introducing coaching program goals and the faculty coach role, in addition to future considerations around group coaching opportunities.

## 1 Introduction

Learners in graduate medical education (GME) face several unique challenges of their stage of training which may hinder their ability to independently achieve their professional potential [1]. The transition from a regimented educational experience in medical school to a more fluid and variable learning environment in residency can make it difficult for trainees to self‐identify learning objectives that will serve them long‐term, find and correct gaps in their medical knowledge and skill, and determine effective learning strategies for their careers, all on top of the rigors of clinical schedules during residency training [1, 2]. A paucity of dedicated education and experience in learner self‐reflection and the creation of individualized learning plans (ILPs) further complicates the independent learner's growth and development [1, 3].

Faculty physicians often take on the responsibility of supporting their learners through these professional struggles to help them achieve individual goals and success. Historically, this support is offered through the role of an advisor or mentor, both of which have been the cornerstone of medical learners' growth and development for decades. While there remains a critical need for mentors and advisors in a learner's trajectory, both of these roles embody a more hierarchical relationship structure that may prohibit a learner from being truly vulnerable and honest for fear of disappointing someone whom they respect or the perceived risk of repercussions [2, 4]. Furthermore, the more directive, faculty‐mediated approach in these roles limits the ability to account for each learner's specific situation, struggles, motivations, and needs [4]. This may lead to a disconnect between what the advisor/mentor suggests as a solution and what the learner truly needs or desires.

Coaching intentionally minimizes the hierarchy between faculty and learner by emphasizing a partnership built on trust and safety, opening up an environment to encourage learner self‐reflection and self‐regulation [2]. Coaching particularly differs from advising and mentoring in that it incorporates a behavioral approach that is more curious than directive and embraces a growth mindset, allowing for a more bi‐directional exchange and relationship dynamic that deliberately centers around the learner [2, 4]. Coaching prioritizes psychological safety to allow learners to be vulnerable and open in order to truly explore opportunities for growth, create goals that are meaningful to them, and develop individual accountability for progress and self‐assessment [2]. A coach helps facilitate the learning and implementation of these crucial skills over the course of training, and through this tailored coaching experience, encourages the development of the master adaptive learner who is able to self‐regulate over time until expertise is achieved [5]. The development of these skills is critical for preparing GME learners for independent practice even beyond training.

Coaching is increasingly identified as a valuable component of the competency‐based medical education (CBME) framework which focuses on competency achievement over a time‐based curriculum [6]. Coaching addresses one of the five core components of CBME, “competency‐focused instruction,” which is intended to individualize teaching based on the learner's foundational start and pace [7]. The tenets of coaching allow for programs to focus on each individual learner within the broader curriculum, and in conjunction with medical education tools such as ILPs [8], learner progress can be intentionally tracked towards competency achievement. Coaching has been incorporated successfully within the Canadian CBME model [9], and continues to hold great promise in initiatives within Emergency Medicine [10].

Emerging literature demonstrates that coaching can improve GME learners' workplace well‐being metrics [11, 12, 13], technical skills performance and procedural confidence [14, 15, 16], and communication [17, 18, 19]. Coaching has been shown to improve learner self‐reflection and utilization of feedback, with learners noting that coaching helped them be more proactive in identifying gaps and actively seeking out feedback to gather more objective data [20]. Residents also note that they have little to no experience with goal creation prior to residency [21], with many having negative perceptions of goal‐setting as it can feel forced or too vague [1]. Coaching can help focus resident learning by encouraging more intentionality with learning goals that give them forward movement [21]. However, the majority of these results are obtained via analysis of survey data. There remains a paucity of literature that assesses in‐depth, narrative learner perceptions of longitudinal coaching approaches in GME [22, 23, 24, 25, 26, 27].

Because coaching requires significant buy‐in from the learner [2], there remains a critical need to understand what learners may need and want from the coaching approach, and furthermore, to optimize how coaching initiatives are being adapted to their unique needs. The objective of this manuscript is to highlight learner perceptions of a longitudinal coaching program, particularly focusing on qualitative resident feedback obtained through focus group discussions.

## 2 Methods

### 2.1 Goals and Program Development

We implemented a resident coaching program during the 2023–2024 academic year at a single 3‐year emergency medicine (EM) residency program associated with an urban academic Emergency Department in the Midwestern United States. At the time of implementation of this program, the residency program offered 13 first‐year positions for a total of 39 residents. The initial rollout included pairing each individual resident with one of six faculty coaches.

This coaching program was conceptualized to address two overarching concerns that were identified from our annual ACGME and institutional surveys: a decrease in resident satisfaction with faculty members' feedback and an increase in self‐reported burnout. The goals of this longitudinal coaching program were to improve residents' ability to reflect on and subsequently act on feedback with self‐directed plans for improvement, build individual resident skills for lifelong learning, promote overall professional development, and foster resident‐faculty interactions by growing longitudinal relationships. While the primary focus of the coaching relationship was to focus on professional goal development, both faculty coaches and residents were strongly encouraged at the initial program introduction to consider a more holistic and supportive approach to coaching that included personal goal development as well. Faculty were then encouraged to create a welcoming environment and check in with residents overall during quarterly meetings, recognizing that residents may not choose to discuss their personal lives with their coaches.

The coaching program was initiated in place of a pre‐existing mentoring program in which each incoming first‐year resident was paired with a faculty mentor in their self‐reported, current area of interest or perceived compatibility as determined by the residency leadership team.

Additional details of the coaching program development, faculty coach training, and logistical rollout are available in Table 1.

**TABLE 1:** Design and implementation of a longitudinal coaching program.

Coach selection	The Director of Resident Coaching (SKM) was an Assistant Program Director at the time of program development with an interest in coaching in GME. Although consideration was given to the inherent conflict of using internal faculty as resident coaches [22], alternative approaches such as using professionally‐trained coaches or external faculty coaches from other departments were fraught with logistical challenges (e.g., recruitment, scheduling conflicts, cost). Thus, six additional internal faculty members volunteered to join the inaugural coach cohort after a department‐wide solicitation. All coaches were core faculty with varying roles in medical education (including Assistant Director of Medical Student Education, Director of Medical Student Education, Director of Simulation, Director of Quality Improvement Curriculum for Residents, Fellowship Director for Medical Education, and Medical Education Fellow). The coach cohort consisted of one fellow, four Assistant Professors, and one Associate Professor. SKM did not participate as a coach the inaugural year given her role as Director of Resident Coaching may have been perceived as a conflict of interest by residents.
Coach‐resident matching	Second‐year (PGY2) and third‐year (PGY3) residents who had already been a part of the pre‐existing mentorship program were intentionally paired with a faculty coach who had previously been their mentor to maintain longitudinal relationships that had already formed. These particular faculty who were reassigned from mentor to coach were only expected to meet with these residents in their new role as coach. The remaining second‐ and third‐year residents had mentors external to the selected coaches and thus were randomly assigned a faculty coach. The 13 first‐year (PGY1) residents had randomly assigned coaches rather than assigned mentors as a part of this new program.
Coach training	The coaches underwent faculty development training over the course of several months. This longitudinal faculty development curriculum has been published previously and included a multi‐pronged approach to introduce coaching principles and communication/relational skills through asynchronous and synchronous sessions [23]. As coaches did not receive the degree of training required for official coaching certification [24], but instead learned critical foundational aspects of coaching to incorporate into interactions with residents [2, 25, 26], this learned approach was considered the “coaching approach” rather than coaching in the traditional sense. This is similar to internal faculty training approaches used in other GME coaching programs [22].
Coaching sessions	As a main focus of the coaching program was improving use of resident feedback, individual 1:1 coaching meetings coincided with quarterly Clinical Competency Committee (CCC) feedback dissemination. Topics related to personal wellbeing and work‐life integration were incorporated into these discussions as desired by the resident. Residents were advised to meet with their coach quarterly and schedule additional meetings as needed. The SMART goal [27] approach was used to inform ILPs (Appendix B) that were strongly encouraged to be documented on a template provided to coaches to work on with their residents.
Coach families	Each coach was encouraged to organize or attend at least one social gathering with their assigned residents in order to strengthen longitudinal relationships between coaches and residents, as well as between residents, to allow near‐peer mentoring. Each coach was allocated $300 of departmental funds for coaching family activities in the inaugural year.

This project was structured as a qualitative process evaluation conducted at the end of the inaugural year of the coaching program. It used focus group interviews to collect resident perspectives on their experiences with the introduction and implementation of coaching in place of assigned mentoring.

The semi‐structured interview guide was initially developed by the Director of the Coaching Program/lead author. It was iteratively refined after discussion with senior medical education faculty in the department. Additionally, it was presented at our monthly education research meeting where further refinements were made.

### 2.2 Data Collection

Per our University's IRB policy, this project did not meet requirements for formal IRB review as it was viewed as quality improvement/program evaluation.

Resident focus groups were conducted at the end of the 2024 academic year (in June and August) to gather qualitative data to inform a broader program evaluation. Focus groups were conducted by authors CTM and BHS using a semi‐structured interview guide (Appendix A) focusing on programmatic areas of strength and opportunities for growth with the purpose of revising the program to better fit resident needs. Each focus group was composed of residents from a single post‐graduate year (PGY) which allowed residents who were introduced to the coaching approach at the same stage of residency training to discuss their experiences with each other. Four focus groups were planned based on each resident's post‐graduate year during the 2023–2024 academic year: 2 for the PGY3 class (to accommodate scheduling challenges), 1 for the PGY2 class, and 1 for the PGY1 class. These focus groups were scheduled for 60 min, but could be concluded early/late depending on the discussion from the participants. Focus groups were audio recorded using Voice Memo (Apple, Cupertino, CA) to allow for anonymized review of the transcript to assess for themes.

Residents were invited to attend focus groups which occurred during weekly residency didactic time. Participants who elected to attend were made aware that the session was being recorded and that their responses or non‐responses would not affect their status within the program in any way. Additionally, focus group facilitators were chosen based on their lack of direct involvement in the coaching program and residency leadership team to avoid biasing focus group responses. Participants were also free to leave at any time and were given alternative options for sharing any feedback that they were not comfortable sharing in a group setting.

### 2.3 Data Analysis

All of the recorded audio data was transcribed using Otter.ai (Otter.ai, Mountain View, CA). This study utilized a constructivist thematic analysis methodology [28], following an inductive approach to generate themes directly from the data. The written transcripts were personally reviewed by the lead author to verify accuracy and correct speaker assignments. These finalized transcriptions from each of the four focus groups were then preliminarily coded by three authors (CMJ, DEL, MSA) independently. During the preliminary coding, the three authors each developed their own initial codebook and then met to synthesize and revise a joint codebook. Codes were then organized into broader categories and themes. The joint codebook was then presented to the full study team. All codes and themes were reviewed and critically discussed as a group. Additional refinements were made following this discussion and a final codebook was created. After review of this final codebook, the team agreed that all perspectives present in the focus groups were well‐represented and saturation had been reached with respect to content. As such, no additional data collection or analysis was performed.

### 2.4 Reflexivity Statement

SKM considered how her role in the residency coaching program might influence how questions were framed and received when she created the focus group facilitator guide. It is also possible that using authors CTM and BHS as facilitators may have influenced how the residents responded to focus group questions given the pre‐existing relationships between these faculty and residents. Additionally, the author cohort included faculty coaches and the Director of Resident Coaching, which may have impacted how the data were interpreted and presented. However, steps were taken, where possible, to minimize bias, and we feel that the manuscript portrays the data accurately. Three separate authors individually completed the initial data analysis to incorporate investigator triangulation, developed a clear and systematic coding procedure, completed peer debriefing with the remaining authors who were not involved in the initial coding process, and outlined the appropriate limitations of the author involvement in data analysis.

## 3 Results

A total of 25/39 (64%) residents participated in the focus groups (8 participants from PGY1, 8 from PGY2, 9 from PGY3). We identified five overarching themes: (1) learner understanding of the coach's role, (2) logistical elements that can affect the effectiveness of the coaching relationship, (3) relational factors that contribute to a successful coaching relationship, (4) learner perceptions of discussion topics and goal creation, and (5) future group opportunities for fostering more personal relationships and professional development.

### 3.1 Overarching Theme #1: Residents Had Mismatched Expectations of the Faculty Coach Role

While many residents acknowledged the multiple roles that academic faculty physicians hold during their training, they had wide variability in their expectations of the faculty coach role. While one resident thought “‘*oh sweet, I'm gonna get a faculty friend*’” (*PGY 1, Resident 5*), another equated academic coaching to sports coaching:

A coach for a sport is going to tell you: ‘this is how you need to run this route. This is how you need to perform this maneuver’…I don't think that's happening in the coaching right now (PGY2, Resident 8).

The most prevalent point of confusion was specifically around the role of a coach and how it differed from that of a faculty mentor. Compounding this uncertainty was that some residents had previously‐established mentors who had become their coaches at the start of this program, and the duality of the role made it a challenging transition.

The issue was my coach and my mentor were the same person, and so that would make it really, really confusing. Like, when are you my coach? And when are you my mentor? (PGY2, Resident 6).

While one resident noted that it would be “*maximum benefit to have your coach be in the same area of interest*” (*PGY1, Resident 6*), another enjoyed having their mentor separate from their coach as they could serve very different roles in their professional development.

Lastly, residents identified a need for coaches to be clear on their own role, with one noting: *“I just want to emphasize the point that was made about teaching the coaches how to coach. I think that's going to be really important moving forward, and making sure that that's ongoing”* (*PGY2, Resident 4*). They perceived the uncertainty and lack of clarity around the coaching role is likely in part due to the need for ongoing faculty development for the coaches.

### 3.2 Overarching Theme #2: Logistical Elements That Can Affect the Effectiveness of the Coaching Relationship

#### 3.2.1 Subtheme #1: Timing of the Coaching Introduction During Training Can Determine the Effectiveness of the Longitudinal Coaching Relationship

Residents voiced a common belief that introducing the coaching program earlier in residency training would be most beneficial to promote a welcoming environment, allow for more continuity in the coaching relationship, and provide a maximized opportunity for professional development.

It's really nice to have a faculty from the beginning that's invested in my success and interested in me…and having it from the beginning when you're very much lost and don't know anybody, don't know who to reach out to, is really helpful (PGY1, Resident 7).

Some of the perceived downsides of starting coaching later in training was that the senior residents had already built relationships with many of these faculty members and they had difficulty viewing them in a new role. There was also an overarching sentiment of being “done” and having less benefit from the coaching conversations later in training.

I feel like most of my goals were already figured out by the time we started because I had already been applying to fellowship. I think most of us were at that point…So it would've been much more helpful intern year (PGY3, Resident 9).

One junior resident noted, however, that having insight into future goals and direction might be difficult as a new resident, noting that. *“*…*perhaps [I was] not as willing to engage in those conversations about what I want out of my career when I'm just starting residency*…*that overwhelmed me”* (*PGY1, Resident 6*).

#### 3.2.2 Subtheme #2: There Needs to Be a Balance Between Meeting Frequency With the Coach and the Logistical/Time Burden

Residents reported that scheduling coaching meetings did not add “*an undue burden*” (*PGY1, Resident 1*). In fact, one resident felt that there was a minimum amount of meetings needed in order to build a relationship with their coach.

I think it's the balance of time commitment, right? Because I don't want to be meeting all the time with my coach, but meeting twice a year…is not enough to build rapport, or just build that trust (PGY1, Resident 6).

Another resident commented that increasing meeting frequency might establish a strong enough relationship to overcome any preconceived apprehension of coach pair matching.

When asked which format they preferred for meeting with their coach, one resident noted:

I think you definitely needed to meet in person. You cannot actually have conversation over email…especially for what I think is intended from this program. Talking about ways in making that [feedback] actionable and specific goals needs to be an in‐person kind of conversation (PGY1, Resident 2).

#### 3.2.3 Subtheme #3: Residents Desire a More Structured, Coach‐Driven Meeting Series

The majority of residents felt that a more structured meeting format and agenda, with clear and dedicated objectives and topics to cover per session, would be most useful for the coaching program.

They also desired a more coach‐driven discussion and relationship.

I feel like it should come from the coaches…I feel like they're trying to put it on us and it's not going to happen if it's put on us because we have too many other things that we're trying to survive with (PGY2, Resident 1).

As stated above, many residents' understanding and perceptions of the coach role in this new program were mirrored from their prior experience with sports coaching, which takes more of a coach‐led approach.

Some junior residents noted that coaching conversations are unfamiliar and can be difficult:

I'm new at this. I don't know [how] to do [it]…I know that they were trying to get me to think about it, but I'm not informed well enough to know what things I should be working on and how to do it (PGY1, Resident 7).

Another noted:

My coach has been very much invested in making me figure out what I'm going to do about it, instead of telling you what to do, which I'm sure is well‐founded in some research, but I don't like it. I want you to tell me how I can do that (PGY1, Resident 6).

One resident posited that more structure and guidance early in the coaching program would allow them to “*get the feel of what coaching is supposed to be like at the beginning of the year”* (*PGY2, Resident 6*).

### 3.3 Overarching Theme #3: Relational Factors That Contribute to a Successful Coaching Relationship

#### 3.3.1 Subtheme #1: Residents See Value in Pairing With Faculty Coaches Who They Perceive Have Relatedness and Credibility

Residents had variable opinions on whether or not utilizing internal faculty as coaches was beneficial. One noted that “*historically you get coaches who are not in your specialty so that you can actually have a relationship that is based purely on coaching*” (*PGY3, Resident 3*). Another noted that having someone who knows their clinical environment and department was beneficial for problem‐solving certain issues.

Most residents saw more value in pairing with a coach who demonstrated some degree of relatedness. One resident “*enjoyed that my coach was more of a young professional than my previous mentor. So I think it made it significantly easier to connect…[they] were really relatable to where we are right now”* (*PGY3, Resident 8*). Others voiced value in having some personal and professional connectivity:

There's some value to having someone who you were closer with…you value their opinion, perhaps to a higher level, just because they seem to like the things that you like, or want to do things that you like, or you want to model your career after theirs. And that's tough to do when it's someone pre‐selected (PGY1, Resident 2).

Another important factor was viewing the coach as a credible source, and that this was necessary in helping residents improve upon areas for growth.

What feels just off about the coaching situation is I want specific tasks and things that I feel weak in to be able to get better at that. So the coach that I was assigned is great, but it's not one of the faculty that I specifically feel can augment my weaknesses (PGY2, Resident 8).

Lastly, several residents noted that there are some scenarios in which the assigned coach pairing may just be a poor fit despite putting in the effort to build a successful relationship. They note it is imperative to have a psychologically safe option to transition coaches when such scenarios arise:

I think there should be an avenue, if you get to the point, ‘I don't think that this relationship is working,’ to be able to find a way to just press the eject button (PGY1, Resident 7).

#### 3.3.2 Subtheme #2: Developing Relational Trust and Safety Allows Residents to Be Open and Vulnerable

Residents varied in how comfortable they felt in the coaching relationship in order to open up about personal or professional struggles. One resident positively commented on their relationship with their coach:

I love my coach, we have a great personal relationship outside of coaching. I trust them wholeheartedly…But at the same time, if I had a different coach I might not actually share personal things with them right because there is potential professional consequence (PGY3, Resident 3).

Another commented:

There are issues with just getting assigned to somebody who might not be a person that you are willing to have very open discussions with (PGY3, Resident 4).

Of residents who felt comfortable discussing a wide array of topics with their coach, they describe their coaching experiences by using phrases including “*protected and safe*,” “*I didn't feel like anything I was gonna say would leave the walls of our conversation*”, “*cathartic*,” and “*comfortable.”*

### 3.4 Overarching Theme #4: Resident Perceptions of Discussion Topics and Goal Creation

#### 3.4.1 Subtheme #1: Coaches May Help Contextualize Learner Feedback and Give It Credibility

There were many complexities to whether or not coaches were perceived as helpful in contextualizing Clinical Competency Committee (CCC) feedback that residents received.

I feel like my coach was definitely more in‐tune with what my feedback was, and areas of strengths and weaknesses, which is something that I did not have first and second year (PGY3, Resident 8).

Many residents noted that working clinically with their coach subsequently helped give credibility to feedback discussions given the coach had contextual knowledge of the resident's goals and growth trajectory. Of residents who did not work with their coaches, many felt the coaches “*don't have any more insight into the feedback”* (*PGY1, Resident 2*) than they did, identifying a need for higher quality feedback as an input into coaching discussions to yield better conversation.

One resident noted how imperative it is to have rapport prior to receiving constructive feedback, and how a familiar coach can help with that:

I don't want a faceless person I've never worked with…They need to actually see how I'm doing for it to have some credibility, and then I want to have some rapport with them. So when they tell me something that is in a blind spot that catches me off guard, I feel comfortable probing that to make sure I understand what I can work on, rather than just being like, ‘you don't know me’ and shutting it down (PGY1, Resident 1).

#### 3.4.2 Subtheme #2: Residents Voiced a Desire for More Integrated/Holistic Coaching

While the primary goal of the coaching program focused on professional development and feedback utility, residents voiced an interest in a more holistic approach to coaching on a wider array of topics.

So maybe coaching isn't about SMART goals. Maybe coaching is just about general life wellness and mentorship, I think that would make it a more productive thing (PGY3, Resident 4).

Another resident noted how unique of an opportunity it was to have that space with their coach to discuss topics that may not otherwise come up organically with a faculty member.

We had a useful discussion about overall career goal[s]…that was a worthwhile conversation because it's not something that's super easy to have with faculty after shift [when] talking about other goals (PGY1, Resident 1).

#### 3.4.3 Subtheme #3: Residents Had Different Opinions on the Utility of Goal Creation

Some residents noted that goal co‐creation with their coach helped with individual accountability.

I think they did make me think about those greater goals, because on the day to day, you're just just trying to make it through things, and there's a lot less ‘what do I want to progress on over the year? What do I want my career to look like?’ Which are broader questions that I have avoided because they're big and scary. It's easier to just go on shifts. So I think that has made me think about those (PGY1, Resident 2).

I feel like it's helped me stay accountable. Like, one of the goals was, ‘oh, I want to start flying’. I was like, ‘I haven't taken any steps to do that’. And so it's like the goal in the next six months is to email…and I feel good about myself (PGY1, Resident 5).

Those that thought SMART goals weren't helpful or had a general disinterest in goal‐creation believed they were just an additional task to complete among all the other stressors of residency.

[I'm] still trying to study, still trying to maintain some sort of sense of sanity, that having other goals other than survive and thrive during residency and like maintain good mental health, maintain good physical health, like is really asking more than what should be expected of a resident. Right. So I feel like it's interesting to talk about SMART goals but it's much more realistic once you're in a more professional setting like outside of your like graduate medical education (PGY3, Resident 3).

### 3.5 Overarching Theme #5: Future Group Opportunities for Fostering More Personal Relationships and Professional Development

#### 3.5.1 Subtheme #1: Residents Appreciated the “Coach Families” for the Personal Relationships and Near‐Peer Mentoring Opportunities

The majority of residents valued the opportunity to create more intimate relationships with the other residents within their “coach family.”

It just builds relationship between an extra group of people, not just one person. It makes you feel more like there's purpose to all of this. It makes you feel more like part of a family (PGY2, Resident 1).

Some senior residents viewed less benefit from this opportunity as they progressed in their training. However, of those that held this view, the majority still acknowledged the benefit this opportunity held for junior residents in building a sense of community and near‐peer mentorship.

One resident felt that there was a risk of forcing social interactions on residents who did not want to participate:

If the people are not social in the first place, they're probably not gonna like these [family] events…I don't really see the value of all of us getting together, because we're kind of filling those social needs externally, organically, that if you're an introvert, you're not going to show up (PGY2, Resident 7).

#### 3.5.2 Subtheme #2: Residents Identified the Potential for Professional and Clinical Development With “Coach Families”

Residents noted there was an unexplored opportunity to incorporate more team‐building exercises, and possibly group coaching, into the formalized longitudinal program.

We have plenty of organic social stuff. We're really good about that as a residency. But I feel like what we lack is the structured meetings. And so maybe more structured team building sort of meetings might be more helpful, where we have a goal and something to work towards (PGY2, Resident 6).

Another interesting idea was to incorporate “coach families” into the clinical setting.

I think talking about a ‘team’ rather than a ‘family’ makes more sense, right? Because the reason that teams get cohesive is because they are actually doing something externally, right? We can put our coaching teams together in the simulations as best as possible, then we can function like that. And then it facilitates the vertical near peer mentoring, and then the coach gives feedback… I think we're missing that piece right now, and that's where I think teams with coaches function, and that's how they bond, is because there's something that everyone's working towards (PGY2, Resident 8).

## 4 Discussion

GME learners face several different challenges as they transition away from the more structured educational format and evaluative structures of UME. Coaching has the potential to bridge these experiences. Coaching differs from the more common roles of advisor and mentor in that it intentionally minimizes the hierarchy between faculty and resident, promotes psychological safety, and incorporates conversational techniques that are more curious than directive. Coaching holds great potential as a learner‐centered approach in the GME framework, particularly as we prioritize a transition to CBME. However, there remains the need for further research to assess how residents perceive coaching and how we can optimize this approach to fit their needs.

We implemented a longitudinal residency coaching program targeting primarily professional development and incorporating personal development when residents desired. Through focus group discussions, residents note both strengths and areas for improvement for our coaching program, and posit several considerations to focus improvement efforts on for future iterations of the program.

One clear theme concerned the role coaches play for the residents. While the coaching program's explicit goals were fostering professional development, increased self‐reflection on and use of feedback, and promoting overall holistic wellbeing, residents also identified the continued need for mentorship and advising and often looked to their coaches in these areas. This was similarly seen in a study by Baenziger where residents expressed difficulty reconciling role ambiguity while simultaneously desiring a single faculty member to encompass all necessary roles [29]. Another study noted that residents may not have the insight to recognize what roles they need faculty to play at certain times during training [30], which can complicate their expectations. There may also be space for faculty to adjust the role they play with learners along the training journey (advisor/mentor early on in residency and coach later on) [31], or blend the roles together in a single conversation as the scenario warrants [4]. This highlights the need for continued education and clear role delineation for both residents and faculty around coaching, and, if coaches are to primarily focus on performance improvement, ensuring separate career mentorship and advising resources are additionally available to avoid unmet resident needs.

The timing of coaching introduction during residency training was also identified as a critical consideration. The majority of residents saw the value of the coaching relationship and process; however, they also strongly preferred to have the program implemented at the outset of training for optimal benefit in building the coaching relationship and professional development. This is similar to Stroud's results that reported senior residents felt the formalized coaching structure was more of a burden later in training [32]. This may be influenced by the perception that coaching interactions were seen as most beneficial for shaping a learner's trajectory and for those who were “struggling”, both scenarios that senior residents may not view themselves in as they near graduation [32].

Residents clearly valued their coach's perspective and insight on their assessment data, which provided crucial context for their identified areas for growth. Furthermore, most seemed to agree that having faculty coaches to help prompt deeper introspection and self‐analysis was beneficial for identifying gaps in performance and progress. This increased self‐reflection and awareness has been demonstrated in several other GME coaching endeavors as well [32, 33, 34], with residents noting the facilitated discussions allow them to consider big‐picture motivations and goals that they otherwise would not have broached on their own [32].

Despite this increase in self‐reflection, resident opinions differed on the value of goal creation. Those that viewed it favorably noted that creating specific steps to achieve their goals helped give them concrete direction to take, and having a co‐partner for accountability was a useful safety net for progress. Of those that had less favorable opinions, much of the discussion was around the desire for higher‐quality output from CCC to inform coaching discussions to better guide what goals might be useful, rather than a deficit in the coaching conversation itself. Given the wide variety and quantity of assessment data that our residents receive as a part of programmatic assessment, the ability of coaches to add value in this domain is noteworthy. Of note, we did not have sufficient resident commentary to assess successes in achieving the goals they co‐created with their coaches. Some literature notes that despite goal creation and use of ILPs, there remains a gap in residents actually achieving set goals [21]. One study posits that this may be due to a lack of concrete co‐planning during the coaching session [35], while another notes a lack of follow‐up in future coaching meetings to gauge success or address barriers to implementation [33]. Further study is necessary to assess goal achievement within coaching.

One unexpected finding was the strong resident desire for more structured coach‐led discussions. This is contrary to the core of the traditional coaching relationship which is heavily based around client‐centric agendas and discussions [36]. Part of this discordance may be that many residents compared this coaching experience with the more familiar parallel in the sporting world, in which a coach traditionally gives specific feedback and directs areas for focus and improvement. Additionally, the unfamiliarity with coaching may have driven the desire for more guidance at the start of the program; continued experience and comfort with the coaching process may lend to more learner‐driven agendas [34]. Further research is necessary to garner optimal ways to introduce coaching to medical trainees, the differences between academic coaching and sports coaching, and how to engage and empower learners to be more autonomous over the coaching process.

While preference for a closer interpersonal similarity with their coach is mirrored in other studies [11, 29, 32, 33], an unexpected finding was that many residents desired more clinical interactions with their coaches in order to deepen the understanding in the relationship. While this is in contrast to the traditional model of a coach as a detached and non‐evaluative partner [2], it may represent residents' experience with and interest in a sports performance‐coaching model utilizing more direct observation. The direct clinical exposure may also help build the coach's credibility that residents noted as critical to successful relationship development, as seen in another study [37]. Another study noted that residents vary in their preference for having a coach who also supervised them clinically [29], with many noting this as a potential conflict of interest. Future considerations may be to blend our longitudinal coaching approach with the “coaching in the moment” model [9] in order to bring coaching to the clinical environment and provide residents with more of the directed feedback and development they desire.

Lastly, residents identified a desire to further explore coaching group dynamics. Residents seemed particularly interested in the possibilities afforded by having informal families made up of residents with the same coach. While some were mainly interested in the social interactions, others were interested in exploring a group coaching dynamic, which has been successfully piloted in programs addressing workplace wellbeing [12, 38, 39] and professional identity development [40, 41]. This structure may also be conducive to near‐peer coaching models, which have shown positive preliminary results in early studies [42, 43]. While group coaching may provide a more efficient use of a coach's time, careful consideration must be taken when addressing sensitive or personal topics with each individual learner.

This work allowed us to make several important improvements to our longitudinal coaching program to best fit resident needs. All‐coach meetings were held quarterly to continue faculty development; sessions included reviewing pertinent material/articles to further refine and standardize our overall coaching approach in addition to building a shared community to learn from one another's experiences. This also helped continually clarify the coaching role for the faculty and how we could best delineate our roles for residents. There was also a larger emphasis placed on encouraging coach family gatherings in order to foster relationship‐building outside of the clinical setting. Use of departmental funds was streamlined in order to reduce the barrier to coordinating gatherings.

## 5 Limitations

This study had a number of limitations. It was performed within a single academic EM residency program, and therefore the discussion points from the focus groups may be specific to our department and residency program. The focus group format may have elevated or suppressed viewpoints that would have been represented differently in one‐on‐one interviews. While we believe that thematic saturation was reached after multiple focus groups, it is possible that residents who were unable to participate may have had other perspectives that were not well represented here. While faculty coaches were disqualified as potential focus group facilitators, faculty who were familiar to the residents were intentionally recruited in an attempt to create a welcoming and conversational environment; resident discussion may have been skewed by facilitation by faculty within their program. In addition, coaches did not have formal coaching certifications—although this mirrors much of the use of internal faculty coaches in other GME programs [22], this does limit the ability to assess how accurately and effectively coaching techniques were used in resident conversations.

## 6 Conclusion

Residents saw several strengths in the longitudinal coaching approach, particularly around creating a safe environment that encouraged and facilitated feedback reflection, incorporation of feedback into actionable forward progress through goal‐setting, and fostering more accountability for individual progress. There remain areas for improvement when introducing the intent of the coaching approach and expectations of both residents and faculty in the coaching relationship. Future considerations include exploring group coaching opportunities to best serve our residents' interests and needs.

## Author Contributions

Study concept and design (S.K.M., B.H.S.), acquisition of data (C.T.M., B.H.S.), analysis and interpretation of the data (S.K.M., C.M.J., D.E.L., M.S.A.‐M., B.H.S.), drafting of the manuscript (ALL), critical revision of the manuscript for important intellectual content (ALL), statistical expertise (n/a), obtained funding (n/a), administrative, technical, or material support (n/a), study supervision (n/a).

## Conflicts of Interest

The authors declare no conflicts of interest.

## Data Availability

The data that support the findings of this study are available on request from the corresponding author. The data are not publicly available due to privacy or ethical restrictions.

## Appendix A

### Semi‐Structured Focus Group Facilitator Guide

#### Statement of Intent and Confidentiality

First off, I want to assure you that this focus group is confidential. We will be recording this so that we can reflect back and gather themes on areas of strengths and areas for improvement for the coaching program. We will not intentionally collect identifiable information, and should such information be inadvertently collected, it will not be shared or publicized. This information is being gathered to help us perform quality improvement and program evaluation for our coaching program.

Secondly, the intent of this focus group is to tap into your expertise as resident coachees. This coaching program was created solely for the purpose of helping each resident achieve their personal and professional potential during residency training and provide a safe space to reflect, grow, and create goals that matter to you. With that purpose, we are very interested to hear what your experience has been like with the coaching program this past academic year, both good and bad, so that we can continue to improve it to further benefit our current and future residents. We appreciate your perspectives, especially as the senior residents of this inaugural coaching year. If there is feedback or perspectives you'd prefer to share off line, please reach out to XXX, or any other faculty involved in medical education, to discuss in whichever format you'd feel most comfortable doing so.

What has your experience been like with the coaching program this year?

- If mention of feedback:
  ○ How do you feel about the way you have received feedback this year?
  ○ What has your experience been like in discussing your feedback with your coach?
  ○ Has it changed your approach to receiving and reacting to feedback?
- If mention of creating goals:
  ○ How did you feel about creating goals on your own before this program? During and after this program?
  ○ How do you feel this has changed how you will approach creating goals on your own after graduating residency?
- If mention of any type of social gatherings:
  ○ In what ways did this help you or others?
- If any negative experiences:
  ○ What were your expectations for the coaching program?
  ○ What were you hoping the coaching program would be/help you with?

What have you gained from your relationship with your coach?

What were the most effective ways to communicate with your coach?

- Consider the communication of content (feedback) and the most effective format of meeting (in person or text/email)

Are there topics that you discussed with your coach that were most helpful?

- Are there certain topics that you did not like discussing with your coach?
- Are there certain topics that you would be more interested in discussing with your coach?
- How comfortable did you feel addressing personal or professional concerns with your coach?
- How comfortable did you feel in reaching out to your coach with questions or concerns?

What did you enjoy about the coaching program? (What are areas of strength for our coaching program?)

What are some ways that the coaching program could have helped you more this year?

(What are areas for continued improvement for our coaching program?)

Would this program have been helpful earlier in your residency training?

- If yes, how so?
- If no, why not?

What are some ways in which we can further help future residents through this program?

## Appendix B

### Individualized Learning Plan (ILP) Template

Revision Date: ______________

**Table:**

Goal	Desired change	Timeline (begin)	Timeline (end)	Resources required + specific plan of action	Anticipated challenges	Identifiable results
	Describe specific, observable changes that you intend to make after receiving this feedback.					How will you know you have met your goal?
	*What will YOU do?*					How will others know you have met your goal?
1						
2						
3						
